# Development and Initial Validation of the Novel Computational Method for Dynamic Intracardiac Blood Flow Evaluation

**DOI:** 10.3390/diagnostics16091352

**Published:** 2026-04-30

**Authors:** Dmytro Volkov, Dmytro Skoryi, Bogdan Batsak, Iurii Karpenko, Alamjeet Kaur Sidhu, Carola Gianni, Vincenzo Mirko La Fazia, Bryan MacDonald, Joseph Gallinghouse, Rodney Horton, Sanghamitra Mohanty, Andrea Natale

**Affiliations:** 1 Department of Electrophysiology, Texas Cardiac Arrhythmia Institute, St. David’s Medical Center, Austin, TX 78705, USA; 2 Neuron Medical, 63900 Brno, Czech Republic; 3 Department of Innovation Engineering, Private Institution “University of Science, Entrepreneurship, and Technology”, 03113 Kyiv, Ukraine; 4 Department of Development and Innovations, University Clinic of Taras Shevchenko National University, 03113 Kyiv, Ukraine; 5 Departmen of Internal Medicine № 1, Odessa National Medical University, 65082 Odessa, Ukraine; 6 III Medical Faculty for the Training of Domestic and Foreign Students, Kharkiv National Medical University, 61022 Kharkiv, Ukraine; 7 Interventional Electrophysiology, Scripps Clinic, San Diego, CA 92037, USA

**Keywords:** intracardiac blood flow, turbulence, left atrium, atrial fibrillation, echocardiography, cardiac imaging

## Abstract

**Background/Objectives**: To develop and preliminarily evaluate a practical Python-based program for dynamic intracardiac blood flow visualization and the extraction of new quantitative parameters, serving as an initial step toward future flow-based cardiac evaluation. **Methods**: The method was technically explored across five imaging modalities (angiography, MRI, ICE, TEE, TTE) using standard diagnostic hardware. Preliminary testing used ECG-gated ICE DICOM images from sixteen patients undergoing first-time AF ablation. **Results**: The program produced dynamic full-chamber flow visualizations and automatically computed two image-derived surrogate markers of flow-pattern behavior, the *turbulence index (TI)* and *blood mobility fraction (BMF)*, across cardiac cycles. Distinct preliminary flow patterns were observed between sinus rhythm and atrial fibrillation. Outputs are exportable for AI analysis. **Conclusions**: This proof-of-concept approach demonstrates feasibility for routine intracardiac flow assessments, and introduces TI and BMF as potential flow-based biomarkers for future prognostic use after additional validation.

## 1. Introduction

Atrial fibrillation (AF) is an increasingly prevalent global issue, with cases rising sharply in the USA and worldwide. While catheter ablation has become the most effective treatment, 20–30% of patients experience arrhythmia relapses requiring additional interventions [1]. Multi-modality imaging is crucial for predicting ablation success and determining adequate ablation volume [2]. Despite numerous studies and algorithms, accurately calculating thromboembolic risks associated with AF, ablation, cardioversion, and concomitant diseases remains challenging. Investigating and analyzing the blood flow in atrial chambers could provide valuable insights and address gaps in our understanding of AF pathophysiology [3,4]. Some historical and theoretical background on blood flow estimation is provided in S1 [5,6,7,8,9,10,11].

Potential-kinetic energy interchange during flow and blood kinetic energy are crucial, with many measurements and indices describing kinetic energy changes using vector flow mapping and contrast echocardiography via particle image velocimetry [12,13]. Turbulence, with its own features, is an unavoidable result of viscosity during blood propulsion and is connected to blood velocity and kinetic energy. Effects of turbulence can be dynamically traced, measured, and analyzed as an independent factor of blood flow efficiency via different imaging modalities [14,15]. Additionally, calculating energetically different blood pools within the chambers is important for assessing cardiac function and prognosis [16,17]. Recent advancements in blood flow evaluation and calculations offer new prospects for determining multivariate prognoses, including heart failure and stroke [18,19].

Like other chambers, LA flow has been investigated in several studies using contrast TTE and MRI tools. These studies generate various parameters depicting LA flow, with a focus on flow vortex formation and its evaluation as a crucial aspect of blood flow behavior [20,21,22]. Despite significant efforts from multiple scientific groups, a clear understanding of the complex physiological and pathological mechanisms of intracardiac flow remains elusive, particularly in terms of simplified numeric indices. The current study aims to address this gap in a small cohort of patients undergoing their first AF ablation (S2).

## 2. Materials and Methods

**Figure 1:**
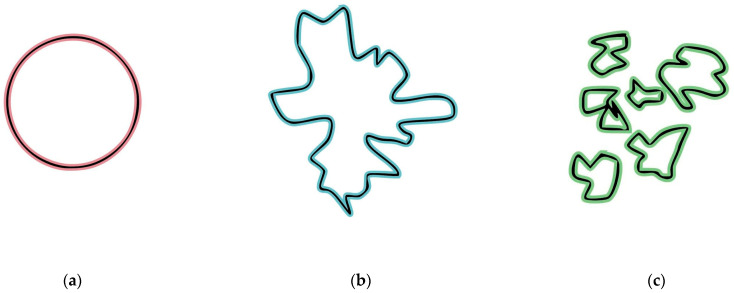
Schematic representation of the different fractal states of moving blood particles as a visual surrogate for turbulence levels: (**a**) a round-shaped object; (**b**) a fractal surface object; and (**c**) disintegration into several highly fractalized separate objects.

As blood flows through cardiac chambers, it inevitably creates some level of turbulence and different moving blood patterns. Visual surrogates of those physical phenomena can be obtained with various radiological diagnostic equipment by special enhancement and digitally represented by dynamic changes of the blood particle dimensions and their fractal characteristics (Figure 1a–c).

The application of a specific dynamic subtraction mode to analyze blood flow patterns, through the continuous digital evaluation of the rate and degree of blood pool fragmentation, is one of the core concepts of the proposed technology. The dynamically subtracted frames are created by calculating the difference in pixel brightness between corresponding points in two consecutive frames. The resulting image represents the grayscale level of the displacement vector amplitude, where black and white indicate the most movable areas, and gray indicates the most stable. A series (cine loop) of these dynamically subtracted frames was used to identify the ‘bright’ actively moving components and the gray passively pooling components, which were further separated into several groups according to their intensity (i.e., isophotes) to emphasize dynamic blood flow changes.

A Python-based (v3.10.9), multiplatform desktop application was developed for the visual representation, extraction, and analysis of dynamic blood flow information, as well as outcome analysis in graphical and text formats. The application processes DICOM files containing image sets and corresponding ECG data from ultrasound, MRI, or angiographic sources. The image set is displayed in the application interface, allowing users to delineate ROI for cardiac cavity and wall segmentation. ROI delineation was performed dynamically, with manual adjustments if necessary, applied to each frame throughout the selected frame series for analysis (Figure S1).

**Figure 2:**
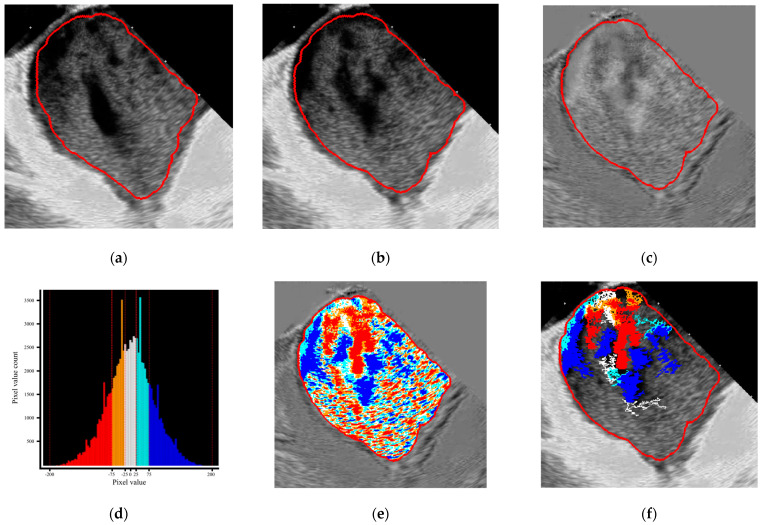
The main algorithm of the raw data elaboration on the example of ICE−derived LA flow: dynamic continuous subtraction of the predefined ROI of two consecutive native frames (**a**,**b**) with the generate on of a differential frame (**c**); color coding of differential frames by dedicated gray scaling (**d**) into 5 color layers (**e**); and programmed pixel cutoff level (200 pixels) to determine the proportion of the pixel size presence above the cutoff (**f**).

The timing of the calculation window is selected based on the ECG strip and can include a defined number of consecutive cycles. Differential frames are then generated by dynamically subtracting two consecutive frames [23]. The resulting image is used to extract grayscale zones (isophotes) with equal intensity ranges for segmentation, color coding, and presentation in per-frame or cine mode. Additionally, the pixel-level cutoff can be determined (e.g., 200 pixels) (Figure 2a–f).

The program has several workflow steps: (1) dynamic subtraction of two consecutive native images; (2) generation of a series of dynamically subtracted frames; (3) color coding of the obtained gray spectrum in five layers; (4) dynamic visualization of the blood flow by choosing active layers; (5) determination of pixels area withdrawal to filter blood particles dimensions; (6) TI and BMF calculation and numeric and graphical representation; and (7) digital data export.

Two new indices of intracardiac blood flow were proposed:

• Turbulence index (TI)—fractality level of the different blood pools;
• Blood mobility fraction (BMF)—pixel dimensions coding of the moving blood particles.

Certain formulas and approaches were used to implement the program’s algorithms and calculate indices, including the creation of the isophote level intervals to structure the image (S3), TI calculations (S4), and BMF calculations (S5).

The current program version limits the number of isophote layers to five, based on grayscale intensity (red and violet—marginal; orange and blue—medial; white—median). The original TI calculation is the relative ratio of the actual pixel contour length to the minimal possible perimeter of a circle with the same area [23]. The program performs both aggregative and layer-based calculations to assess the distribution of isophotes, their absolute area in pixels, their relative area compared to other isophotes, and the overall ROI. It also tracks changes in these parameters per frame, along with overall TI and its per-frame variations. Additionally, the program includes all levels of isophotes for processing, depending on their absolute area in pixels, by establishing a cutoff isophote area threshold. The BMF is calculated as the percentage of the summed pixel area after cutoff relative to the total ROI, representing the most active, “movable” blood clusters. The program’s algorithm flowchart is presented in Figure S2. The program’s working window is presented in Figure S3.

**Figure 3:**
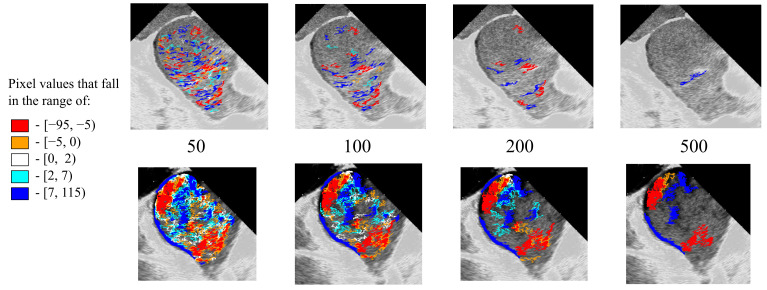
Visual representation of the conduit LA phase in persistent AF (**upper row**) and paroxysmal AF with SR patients (**bottom row**) by incremental pixel cutoff (50-100-200-500).

The observation of smaller square particles during impaired LA flow, possibly representing pooling blood segments, led to the idea of calculating blood particle pixel size as a marker of hemodynamic effectiveness. A higher relative percentage of larger pixel sizes was associated with more active flow, termed blood mobility fraction (BMF). Cutting off smaller pixel size isophotes can enhance blood flow visualization and reduce echo noise (Figure 3).

16 patients (age 66.9 ± 15.2) with their first elected AF ablation procedure (paroxysmal—8 (female—2), persistent—8 (female—4)) were included in the current study. Main basal clinical and instrumental data of the patients are presented in Table 1.

Patients with persistent AF were older, had obesity, more dilated LA, and a higher grade of MR and CHA2DS2VASC score.

All the patients underwent successful, uneventful PVI, roof line, and PWI, except one patient with PVI only. CTI line was performed in 2 patients with coexisting typical atrial flutter. All the patients received uninterrupted direct anticoagulation and were in the therapeutic range of ACT throughout the procedure.

Pulsed wave and color Doppler images of PV and transmitral flow were recorded to characterize the flow and calculate velocities. The Prucka EP system (GE Healthcare, Waukesha, Wisconsin, USA) used ECG measurements. The intraprocedural data are shown in Table 2.

Patients in the persistent group had significantly lower peak velocities in LAA, but higher in RPV and transmitral flow.

ICE ECG-gated DICOM images were recorded by Acunav Acuson probes from the RA position on a Siemens 2000 echo machine (Siemens Medical Solutions, Boston, Atlanta, USA) throughout the procedure. A default frame rate of 80–90 per second and an increased gain of 20 wereimplemented to enhance flow visualization. Then, the DICOM files were used for the offline calculation by the program. Processing load scales linearly with ROI size and frame count. Three consecutive ECG-gated cardiac cycles were analyzed. As a result, the enhanced color-coded visual representation of LA blood flow was outlined, and digital graphs of dynamic TI and pixel number-dependent particles square distributions were created in an ECG-gated LA intervals framework. The obtained color-coded flow information, as well as general charts’ patterns, were delineated visually by a digital comparison of the dynamic TI values and average, maximal, and minimal BMF under different pixel cutoffs (50–1200).

Continuous variables are reported as mean ± standard deviation. Categorical variables are reported as counts and percent. Continuous variables were compared using the Mann–Whitney U test or an independent samples *t*-test, dependent on their distribution. A Chi-square test was used to compare the categorical variable between the groups. All the tests were two-tailed and conducted at an α level of 0.05. A statistical analysis was performed with SPSS statistics (v28.0.1.0) for Windows 25 (IBM, Armonk, NY, USA).

## 3. Results

### 3.1. Visual Representations of the Flow Patterns

The program has the potential to provide a dynamic, whole-chamber visualization of blood flow behavior throughout several cardiac cycles (Figure S4) and subsequently use that information for parameter calculations.

The program was successfully applied in several radiological modalities (Figure S5).

**Figure 4:**
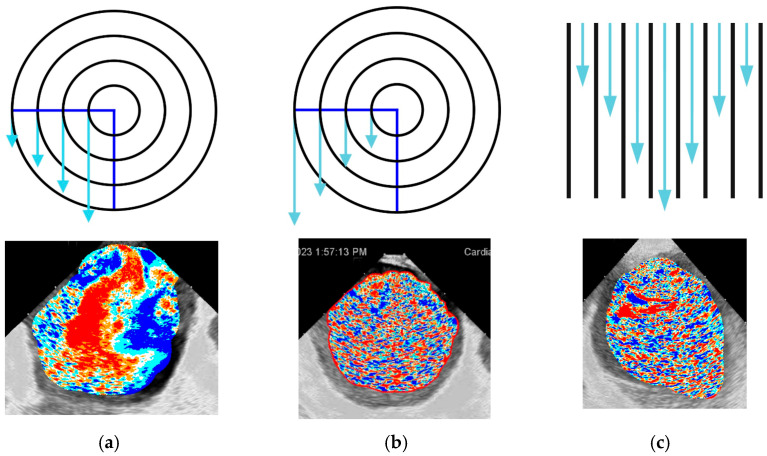
Schematic representation and actual examples of the physical patterns of the (**a**) irrotational, (**b**) rotational vortexes, and (**c**) parallel flow in different patients (Video S1a–c).

The visual effects and patterns of flow created by the software are extremely complex and cannot be directly classified into distinct subtypes. But even in a 2D environment, the program gives a visual enhancement of tracing the main features of the flow, including vortex formation (Figure S6). Nonetheless, as two main types of vortexes, along with parallel flow, exist in classical fluid mechanics, links of actual LA flow to those three models have been attempted. The impaired flow patterns were comparable to those of rigid-body-like vortexes (rotational), which are, by definition, more stable, difficult to change within the cardiac cycle, and need additional energy to support their flow. This usually coincided with mild LA wall contraction and advanced visual dyssynchrony. To some extent, this type of flow was present in all patients with sustained AF, as indicated by the slow, equal-velocity circumflex movement of the blood inside the LA. In contrast, the free vortex (irrotational), which saves initial energy within the vortex, was linked to more visually active flow in patients with initial SR, active synchronous LA wall contraction, and often during flow facilitation under isoprenaline infusion. Parallel flow was present in the regions of severe akinetic LA because of the brisk inward PV flow into the almost completely stuck and unguided LA blood pool, with further slow progression and dissipation. In the worst-case scenario, the flow looked chaotic due to the short-living, small, unpredictable push of blood, with a significant part of the wave-like parallel flow patterns, which is theoretically attributed to the highest stress shear forces (Figure 4a–c).

One more obvious visual conclusion has been made—more active flow in patients with SR and paroxysmal AF was expressed by the larger relative isophote area with active behavior and closer interconnection between isophote clusters. The marginal forms of isophotes were surrounded by medial forms with a thin white median color layer between those groups, indicating the engagement of more passive blood pools by incoming blood flow. In contrast, patients with persistent AF had a more stable, chaotic, and slower flow evolution, ranging from unstable restricted area vortexes, and sometimes consistent whole-chamber slow-moving circles (rotational vortexes), to straight and elliptical, extremely slowly progressing long waves crossing the part of the chamber to become stale without any vortex formation.

**Figure 5:**
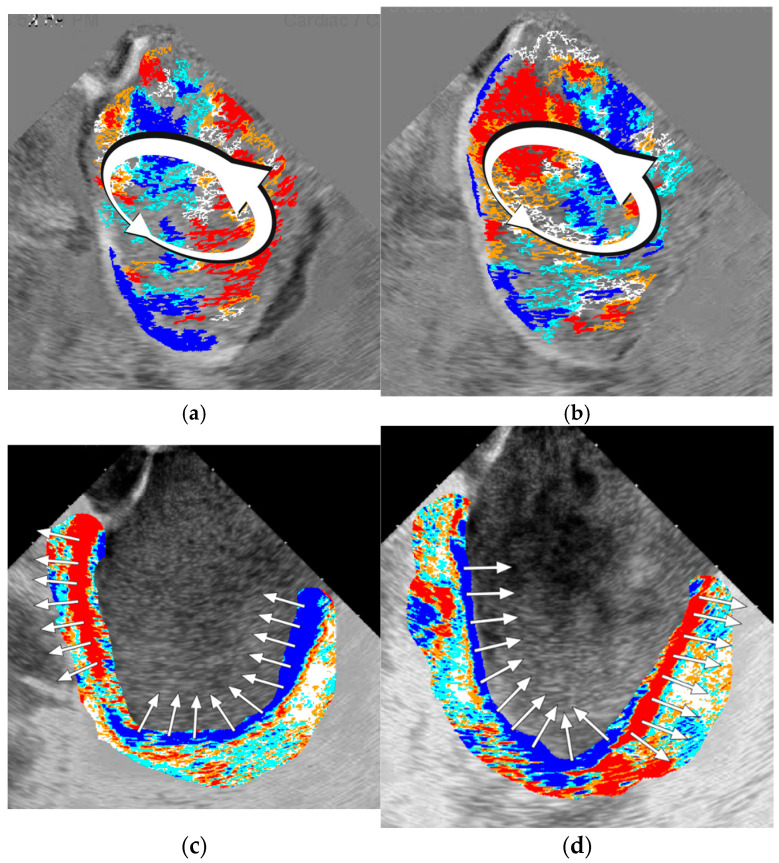
Descending vortex progression parallel to the plane of the MV: (**a**,**b**) two consecutive frames representing the CCW vortex; (**c**,**d**) program color coding of LA walls contraction with obvious dissynchrony in both opposite phases of the cardiac cycle, facilitating the rotational nature of the vortex (Video S2a,b).

These phenomena were inherent to the different ICE projections, including a more anterior LA view closer to the MV with visualization of vortex progression toward the LV, and the contraction pattern of the atrial walls could also be retrieved (Figure 5a–d).

### 3.2. Program-Derived Data Repetitiveness and Correlation with Cardiac Cycle Phases and Other Parameters

**Figure 6:**
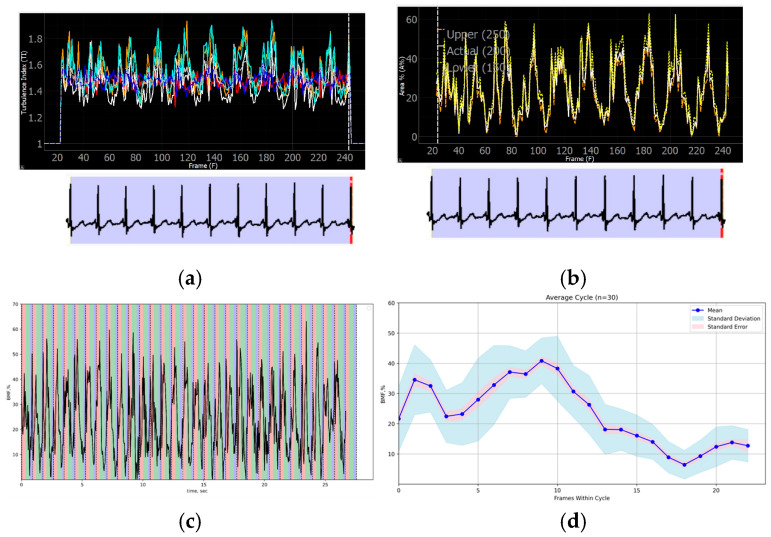
Reproducible program patterns of TI and BMF (200) based on atrial ICE images: 10 cardiac cycles of (**a**) TI curve and (**b**) BMF curve; (**c**) BMF curve of 30 cardiac cycles relative to the reservoir (red), conduit (green) and booster (blue) atrial phases—in addition to cardiac cycle-dependent changes, respiratory fluctuations of the curve can be registered; and (**d**) BMF distribution during the average cycle of 30 consecutive cardiac cycles with standard deviation and standard error relations.

The program evaluation of the ICE-derived data revealed repetitive phased atrial patterns of TI and BMF within up to 30 consecutive cardiac cycles in patients with a stable-rate SR. The preserved atrial mechanical function is presented in Figure 6a–d.

Moreover, the geometry of the BMF curves, for instance, can be distinguished between different atrial pacing sites, reflecting possibly dyssynchronous and alternating blood flow formation in specific circumstances, including cardiac pacing. The TI and BMF curves’ findings are included in S6 (Figures S7–S11).

### 3.3. Comparison of the Blood Flow Patterns Among Study Patients’ Groups

In general, patients with persistent AF exhibited less active and more disorganized blood flow patterns compared to those with paroxysmal AF in SR. The comparative data of the BMF of 200 pixels is presented in Table 3.

The paroxysmal group presents statistically higher maximal BMF 200 values and a greater range between maximal and minimal BMF, indicating more active dynamic flow changes connected to LA phases. Minimal BMF values were comparable between both groups.

**Figure 7:**
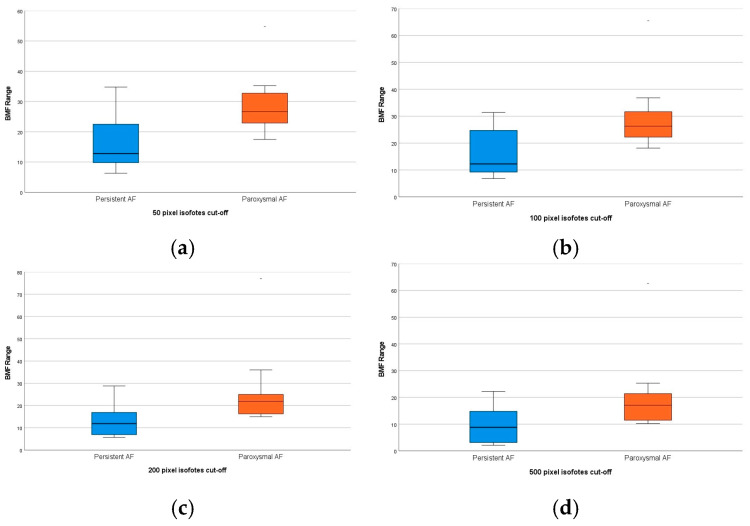
Comparison of different pixel-level (50 (**a**)-100 (**b**)-200 (**c**)-500 (**d**)) average BMFs between groups with paroxysmal. Persistent AF represents statistically higher BMF values in the paroxysmal group.

Patients in the paroxysmal group had a higher average BMF across all cutoff levels (Figure 7a–d).

**Figure 8:**
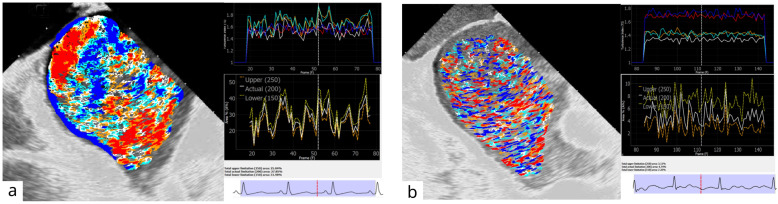
Comparison of visual and graphical blood flow representations in selected patients with SR (**a**) and AF (**b**) from the groups.

A visual and graphical analysis of the program performance of two selected patients with paroxysmal AF in the SR (m, 26; BMI—26.1; CHA2DS2-VASC—0). The persistent AF (m, 73; BMI—30.7; CHA2DS2-VASC—5) is presented in Figure 8a,b.

The abovementioned patients presented obviously different visual flow pictures as well as TI and BMF curves. The TI curve in patients with persistent AF was presented as low-amplitude, rigid, separated lines, and the BMF had chaotic deviations and low absolute maximum and range values. In contrast, the SR patients had more dynamic and synchronized TI and BMF curves, clear connections to the cardiac cycle phases, and several times greater BMF values (BMF (200) 28% vs. 5%, respectively, for the presented comparison).

**Figure 9:**
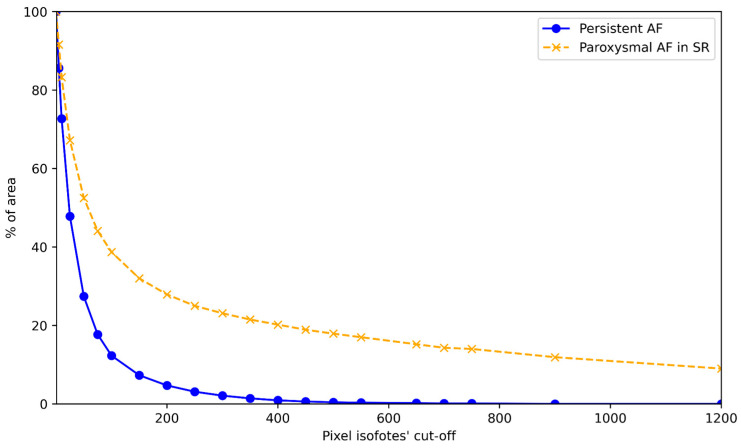
Graphical distribution of max. BMF with incremental pixel cutoff in two selected patients from the persistent and paroxysmal AF groups.

Further analysis of the patients with an incremental pixel cutoff of up to 1200 revealed obviously different graphical representations of the LA flow characteristics, as shown in Figure 9.

## 4. Discussion

Our understanding of cardiac blood flow dynamics has advanced significantly, but many aspects remain to be elucidated, especially considering blood flow as a key indicator of electro-mechanical efficiency. Some researchers suggest that evaluating blood flow might offer a more precise prognostic value than merely tracing cardiac wall displacements [17].

Understanding diverse blood flow patterns and developing new, digitalized indices of dynamic intracardiac blood flow are crucial in the era of personalized medicine, advanced technology, and the machine learning revolution. A dynamic, color-Doppler-like tool applicable to whole-chamber visualization would be valuable to various specialists [11]. Providing digitized information alongside traditional views, especially in a straightforward numeric format, enhances its utility. On the other hand, such a derivative approach can generate specific data sets amenable to AI.

To our knowledge, this study is among the first pilot reports using ICE-guided, non-contrast imaging for dynamic, whole-chamber flow visualization, evaluating and calculating exploratory image-derived surrogate markers from routine clinical images.

The program’s visualization of blood flow revealed its vortex-like nature in the LA, influenced by patients’ rhythm and medical history, consistent with previous studies on cardiac chamber flow [24,25]. However, while vortex formation is a significant observation, it does not ensure an adequate flow pattern. Various types of vortices exist, and despite the development of specific indices to characterize them, these are not yet widely adopted by cardiologists.

Historically, echocardiographic imaging of blood flow has relied on echo-contrast agents to enhance visibility, or on high-frame-rate probes for non-contrast acquisition [26,27]. The proposed approach, utilizing native loops and standard probes, appears more feasible for routine application. At the same time, it should be interpreted as complementary to established flow-assessment methods rather than as a substitute for Doppler echocardiography or 4D flow MRI. Those methods provide velocity-based or volumetric hemodynamic information that was not directly benchmarked in the present pilot study.

Despite years of worldwide CHA2DS2-VASC score adaptation, questions still exist about the additional parameters not considered in the algorithm, including blood flow characteristics themselves and factors that directly influence them [28]. Some authors pointed out that LA structural features and connected flow beyond LAA may affect stroke risk, especially subclinical events [16,18]. There are conflicting data about MR as the additional modifying factor of LA thrombus formation, as a higher grade of MR may facilitate the flow and decrease the probability [29,30]. Calculating LA flow could fill up this potential gap and be used as the missing point of the CHA2DS2-VASC score, as well as an independent stroke risk factor [31,32]. This could enhance thromboembolic risk stratification by digitally quantifying the “smoke” effect not only in LAA, but in LA itself.

We acknowledge that this pilot study to test the software and to preliminarily validate the new approach has clear technical, clinical, and statistical limitations. Image quality dependence is a primary technological concern that can influence results, and the workflow is also sensitive to probe stability, gain settings, dropout, reverberation artifacts, and manual ROI refinement. To improve consistency, we used images retrieved from the same model of echo machine (Siemens SC 2000) with predefined, similar settings. However, extending this technology to different hardware would require calibration and additional testing. The flow was primarily calculated in single-plane 2D models, which are inherently less accurate than 3D environments, so transitioning from pixel- to voxel-derived calculations could be the next step. Additionally, blood viscosity, an important factor in turbulence, was not accounted for in the calculations.

The study was conducted on a small cohort of patients without a healthy comparison group, limiting statistical power and the ability to define normal values for the proposed indices. Further research is needed, including statistically justified studies based on a broader range of clinical markers and extended follow-up with corresponding outcomes in a larger patient cohort. Such studies will help further validate the clinical efficacy of the proposed method, refine the proposed indices, and explore the potential role of AI in data analysis (S7).

## 5. Conclusions

1. A Python-based computer program was created to analyze cyclic intracardiac blood flow phenomena with a focus on the image-derived surrogates of dynamic flow irregularity.
2. The program creates an enhanced, dynamic, and color-coded, whole-chamber visualization of intracardiac blood flow from routine radiological image inputs, and the qualitative feasibility was explored across several imaging modalities.
3. The newly proposed TI and BMF were preliminary validated on a small ICE dataset and should currently be interpreted as exploratory, image-derived surrogate markers of flow behavior that have recognizable patterns and warrant broader technical and clinical validation.

## 6. Patents

Patent US 10,631,811 B2, United States Patent; A61B6/032, A61B8/06, A61B8/0883 Method and system for processing of medical images for generating a prognosis of cardiac function. Dmytro Volkov, Valerij Boyko, Alexander Bakai; appl. No 15/060,970; filed 4 March 2016; issued 28 April 2020.

## Figures and Tables

**Table 1:** Demographic and clinical characteristics of the patients.

Patients, *n* = 16	Paroxysmal AF, *n* = 8	Persistent AF, *n* = 8	*p*-Value
Age	59 ± 19	74 ± 8	0.0551
BMI	26.4	29.3	0.1856
CHA2DS2-VASC score	2.0	3.9	0.0205
LVEF, %	58 ± 7	60 ± 10	0.5971
LA dimensions, mm	40 ± 3.8	43 ± 3.6	0.0523
Mitral max velocity, m/s	0.66 ± 0.25	0.89 ± 0.36	0.0657
Mitral deceleration, ms	198 ± 35	183 ± 28	0.5619
MR, grade	1.1 ± 0.4	1.4 ± 0.7	0.1108

**Table 2:** Intraprocedural data of the patients.

Patients	Paroxysmal AF, n = 8	Persistent AF, n = 8	*p*-Value
ECG with SR			
HR, bpm	62 ± 15	70 ± 17	0.5287
P wave, ms	106 ± 15	104 ± 17	0.7948
PR, ms	151 ± 30	158 ± 23	0.6383
QRS, ms	97 ± 26	90 ± 21	0.5287
QT, ms	412 ± 56	397 + 45	0.9601
Peak velocities (ICE), m/s			
LAA	0.48 ± 0.13	0.3 ± 0.11	0.0106
Left PVs	0.47 ± 0.15	0.36 ± 0.06	0.0929
Right PVs	0.31 ± 0.16	0.4 ± 0.18	0.0404
Transmitral flow	0.46 ± 0.07	0.58 ± 0.1	0.0209
Mean LA pressure	13 ± 5	19 ± 7	0.0930

**Table 3:** Maximum, minimum, and range BMF 200 values in the study groups.

Patients	BMF Maximum Value		BMF Minimum Value		BMF Min-Max Range	
	PesrAF	PAF	PesrAF	PAF	PersAF	PAF
1	15.4	41.8	2.8	5.8	12.6	36
2	17.9	16.3	1.28	0.5	16.6	15.8
3	8.4	30.4	2.7	2.4	5.7	28
4	19.6	21.9	2.4	0	17.2	21.9
5	14.2	78.6	3	1.6	11.2	77
6	9.1	15.9	2.2	0.9	6.9	15
7	8.7	18.7	1.8	1.9	7	16.8
8	29.3	25.1	0.5	3.1	28.8	22
Avg	15.3	31.1	2.1	2	13.3	29.1
SD	7.1	21	0.8	1.8	7.7	20.6
*p*-value	0.0238		0.6383		0.0315	

## Data Availability

Data, including raw data, methods, protocols, and software, will be made available on argued request. For requesting data, please write to the corresponding author.

## Supplementary Materials

The following supporting information can be downloaded at: https://www.mdpi.com/article/10.3390/diagnostics16091352/s1, Supplement Figures S1–S6 with explanations Figure S1: ROI delineation example; Figure S2: The program algorithm flowchart; Figure S3: Program operational working window: (a) choosing DICOM file, selection ROI for the further processing; (b) calculation of differential frames, activation/hiding isophotes layers, adjusting isophote(s) area cutoff with recalculations; (c) choosing imaging modality for processing (MRI, Echo, Angio); (d) imaging window for representing default and processed cardiac images; (e) ECG gating with ROI slider, the play cine button and sliders for image series navigation; (f) data calculation button (g) selection between digitized charts of calculated data; (h) curves series visual representation with active sliding bar of the frames; (i) numerical additional information related to the graphs; (j) digital data export; Figure S4: RA flow visualization with an enhanced TR visualization; Figure S5: Program visualization of blood flow via different imaging modalities: (a) contrast angiography, (b) MRI, (c) TTE, (d) TEE, and (e) ICE; Figure S6: Vortex formation examples: (a) by incoming blood in the conduit LA phase; (b) facilitation of PV flow visualization by 200 pixel size cut-off with one stream from left PVs along the LA roof mixing with vertical stream from right PVs coming along the IAS to the LA bottom forming counter clock vortex; (c) partial clockwise mixed vortex by LA and MR fusion after cardioversion in the patient with persistent AF; (d) complex RA flow pattern by two interconnected vortexes by colliding flow streams from incoming blood and TR. Supplement S1: Historical and theoretical background. Supplement S2: Aims of the study. Supplement S3: isophote level intervals calculations. Supplement S4: TI calculations Supplement S5 BMF calculations. Supplement S6: TI and BMF correlations: Figure S7: Comparable color-coded visual representation of BMF (200) peaks of the flow in 3 consecutive cardiac cycles (a, b, c) with exact connection to the same time-moment on ECG (120 bpm, coronary sinus atrial pacing); Figure S8: LA BMF curves during fast atrial pacing (120 bpm) from CS (a) and low crista terminalis (b); both BMF curves representing peaks occur during LA booster phase but reveal obviously different shapes; Figure S9: Mirrored-like correlation of ICE-derived PW Doppler tracing of the transmitral flow with TI (a) and BMF (200) curves (b) during SR in paroxysmal AF patients (c, e); visual representation of the peak area momentums coincident with the Doppler velocity peaks and associated whole chamber and local vortexes during the conduit and booster phases, respectively (d, f); Figure S10: Comparison of TI and BMF in a patient with persistent AF throughout the procedure in connection with LAA velocities: (a) initial AF; (b) SR after electrical cardioversion, (c) elevated SR under isoprenaline infusion; Figure S11: Dynamic interconnections between curves of TI, BMF, LAP and ECG: (a) patient with paroxysmal AF in SR - close correlation between TI and BMF peaks with pressure curve and ECG gating representing three LA cardiac phases; (b) patient with sustained AF- chaotic pattern of TI and BMF curves, weak correlation with LA pressure and ECG; note lower level of average TI (1.52 vs. 1.59), BMF(200pxl) (4.7% vs. 27.9%) for patient with persistent vs. paroxysmal AF in SR, respectively. Supplemental S7: Final remarks and perspectives. Video S1a: Physical pattern of the irrotational flow; Video S1b: Physical pattern of the rotational vortexes; Video S1c: Physical pattern of the parallel flow; Video S2a: Program color coding representing the CCW vortex; Video S2b: Program color coding of LA walls contraction.

## Abbreviations

The following abbreviations are used in this manuscript:

AF	Atrial fibrillation
AI	Artificial intelligence
BMF (n)	Blood mobility fraction with n-pixel cutoff
CW	Clockwise
CCW	Counterclockwise
ECG	Electrocardiogram
ICE	Intracardiac echocardiography
LA	Left atrium, left atrial
LAA	Left atrial appendage
LV	Left ventricle, left ventricular
MR	Mitral regurgitation
MRI	Magnetic resonance imaging
PAF	Paroxysmal AF
PersAF	Persistent AF
PV(s)	Pulmonary vein(s)
RA	Right atrium, right atrial
ROI	Region of interest
TI	Turbulence index
TTE	Transthoracic echocardiography
TEE	Transesophageal echocardiography
TR	Tricuspid regurgitation
