# Supplementary material for: Development and Initial Validation of the Novel Computational Method for Dynamic Intracardiac Blood Flow Evaluation
Source: Diagnostics (Basel). 2026 Apr 30;16(9):1352. doi: 10.3390/diagnostics16091352 (PMC13163574; doi:10.3390/diagnostics16091352)
Supplement: Supplementary file 1 [file diagnostics-16-01352-s001.zip › Supplement Figures S1-S6 with explanations.pdf]

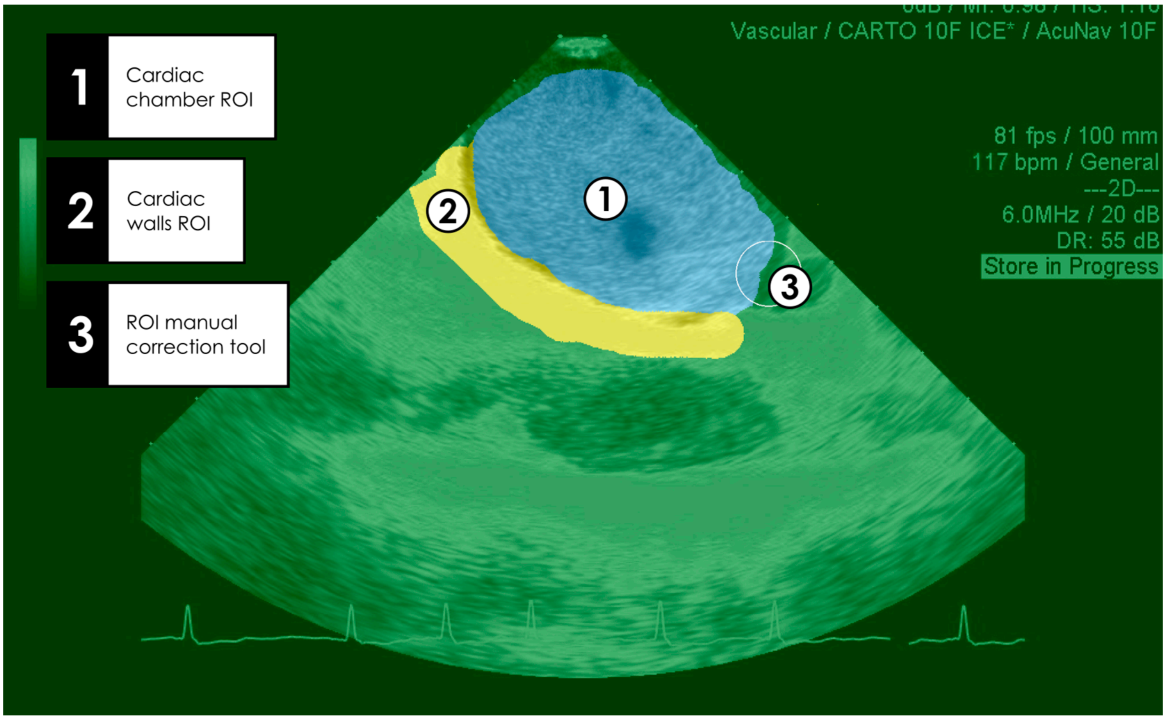

Figure S1. ROI delineation example.

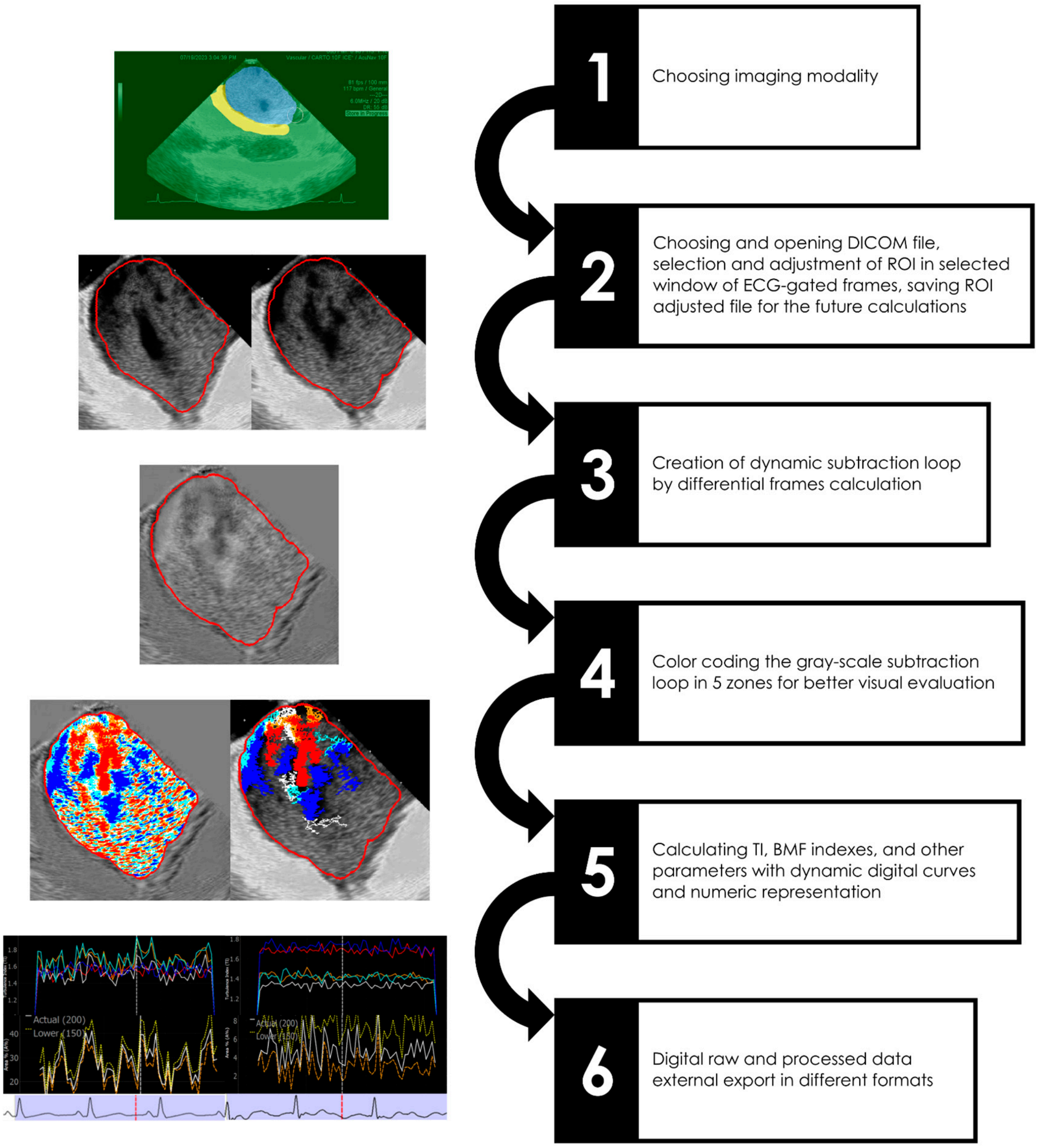

Figure S2. The program algorithm flowchart

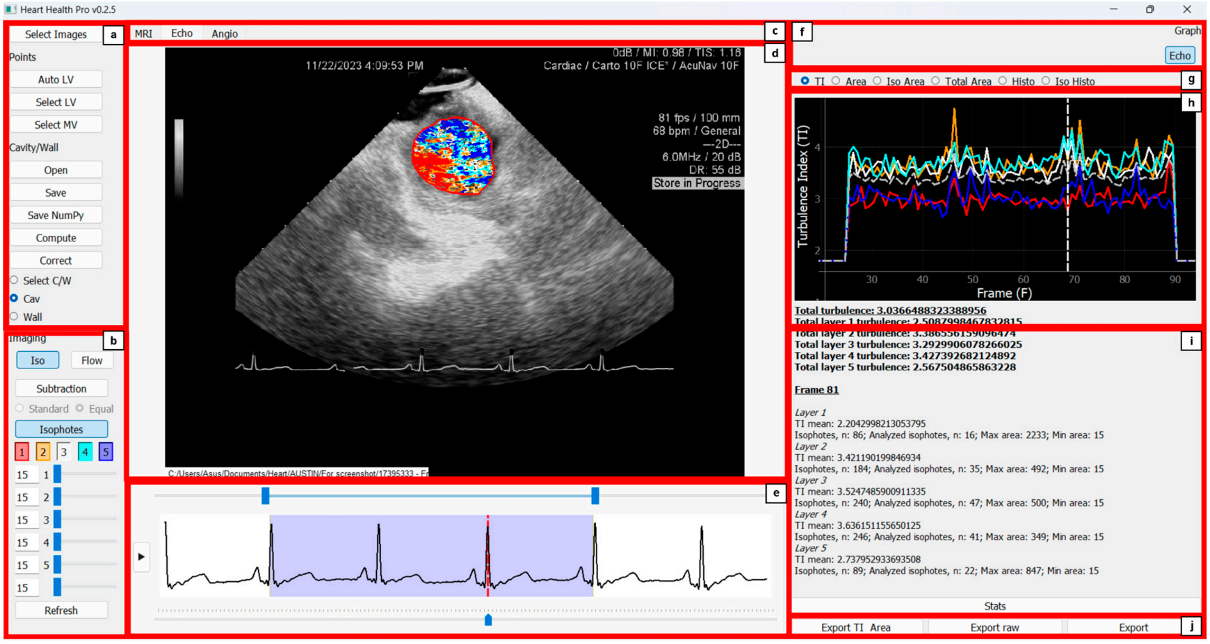

Figure S3. Program operational working window: (a) choosing DICOM file, selection ROI for the further processing; (b) calculation of differential frames, activation/hiding isophotes layers, adjusting isophote(s) area cutoff with recalculations; (c) choosing imaging modality for processing (MRI, Echo, Angio); (d) imaging window for representing default and processed cardiac images; (e) ECG gating with ROI slider, the play cine button and sliders for image series navigation; (f) data calculation button (g) selection between digitized charts of calculated data; (h) curves series visual representation with active sliding bar of the frames; (i) numerical additional information related to the graphs; (j) digital data export.

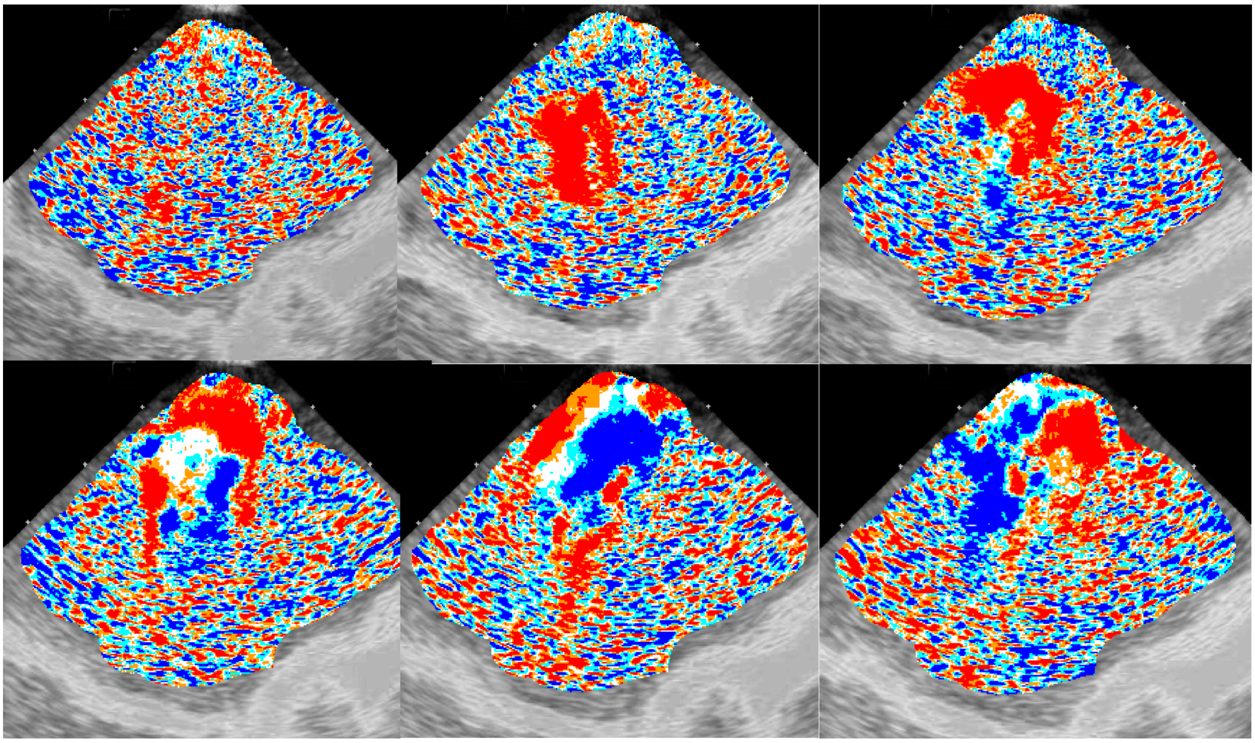

Figure S4 RA flow visualization with an enhanced TR visualization.

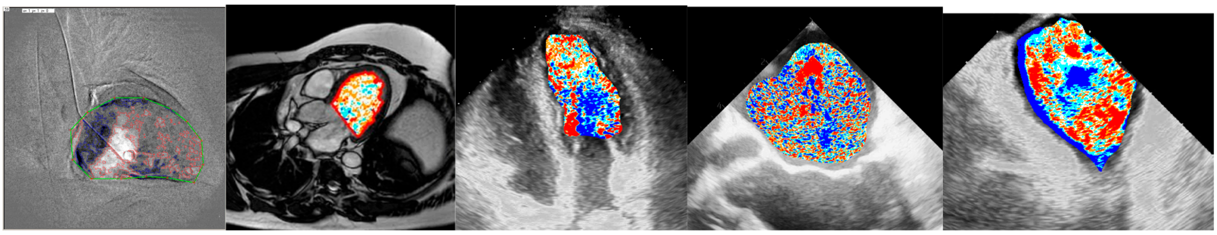

a b c d e

Figure S5. Program visualization of blood flow via different imaging modalities: (a) contrast angiography, (b) MRI, (c) TTE, (d) TEE, and (e) ICE.

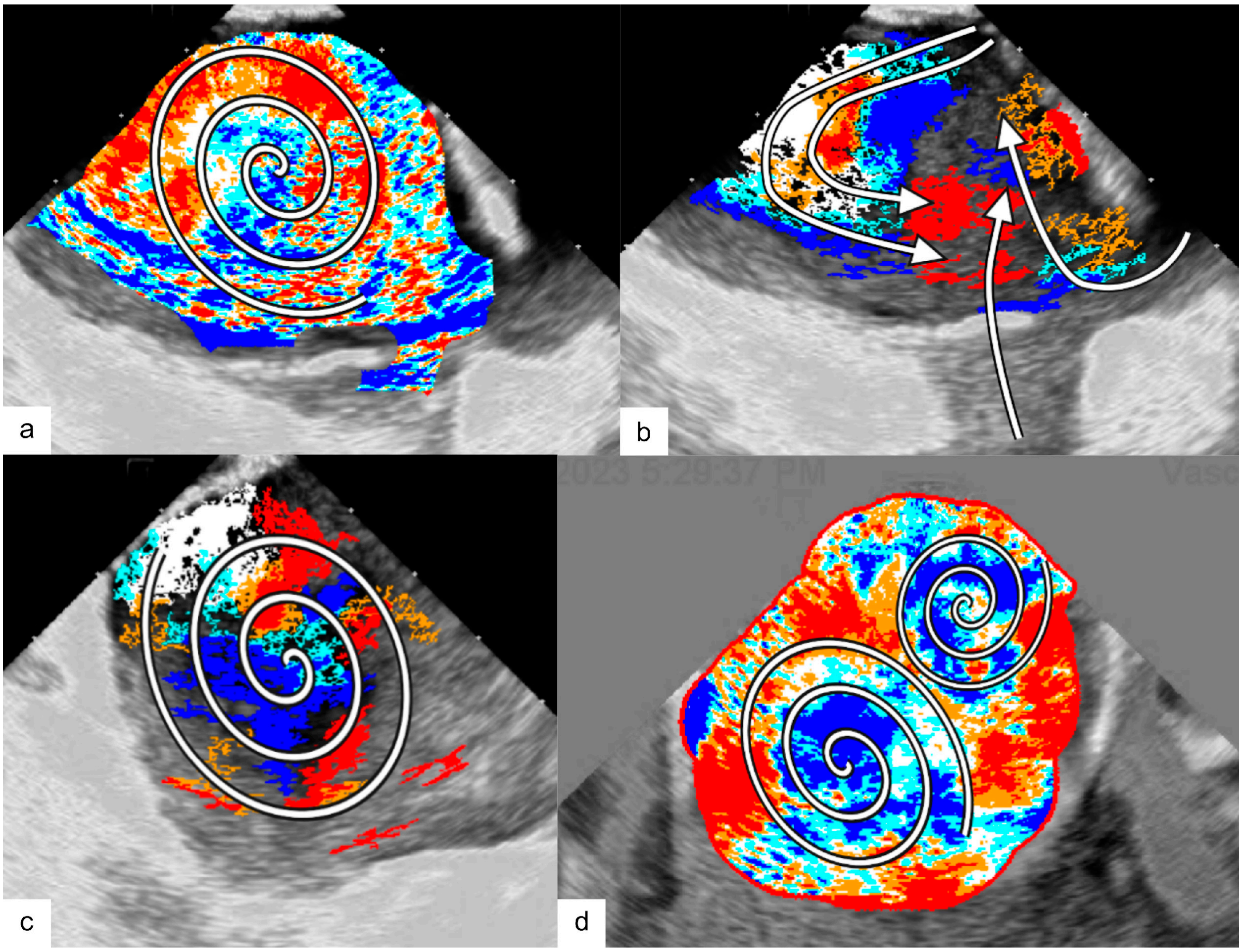

Figure S6. Vortex formation examples: (a) by incoming blood in the conduit LA phase; (b) facilitation of PV flow visualization by 200 pixel size cut-off with one stream from left PVs along the LA roof mixing with vertical stream from right PVs coming along the IAS to the LA bottom forming counter clock vortex; (c) partial clockwise mixed vortex by LA and MR fusion after cardioversion in the patient with persistent AF; (d) complex RA flow pattern by two interconnected vortexes by colliding flow streams from incoming blood and TR.
