# Supplementary material for: Development and Initial Validation of the Novel Computational Method for Dynamic Intracardiac Blood Flow Evaluation
Source: Diagnostics (Basel). 2026 Apr 30;16(9):1352. doi: 10.3390/diagnostics16091352 (PMC13163574; doi:10.3390/diagnostics16091352)
Supplement: Supplementary file 1 [file diagnostics-16-01352-s001.zip › Supplement S1 (Historical and theoretical background).pdf]

Since ancient times, the flow of liquids has intrigued people. Leonardo da Vinci, the first to link this phenomenon to blood circulation, identified over 700 fluid dynamics phenomena and accurately predicted cardiac flow patterns, often depicted as spirals and curls in his artwork. Conventional fluid dynamics, governed by principles like Bernoulli's and Poiseuille's laws, draws parallels with blood flow in cardiac chambers. However, the heart's dynamic, pulsatile nature, combined with valves, blood viscosity, and other factors, complicates these comparisons. It is well known that turbulence in viscous environments increases as flow velocity crosses Reynold's point, though the exact role of this transition is still under investigation. Additionally, the impact of turbulent eddy effects on the macro- and microlayers of flow remains unclear [5].

Different theories address complex flow properties; among them, implementing vortex formations seems to be the most appropriate and accepted for determining intracardiac blood flow. Key models include rigid-body-like (rotational) and free (irrotational) vortexes, as well as parallel flow patterns, which were found to be the most feasible physical models to accommodate within the study [6, 7]. Attempts to create a multilayer picture of cardiovascular functionality employing modern imaging techniques have been made, where fluid flow models can be computed, analyzed, and optimized for different cardiovascular applications [8]. Researchers focus on 3-4D velocity fields of blood flow within cardiac chambers, particularly in the LV, and on calculating specific flow features [9, 10]. New Doppler imaging modalities have been developed for ultrasound [11]. In addition to technically challenging and sophisticated approaches, a few simple and well-known methods, such as specifically designed subtraction techniques, could enhance the expression of dynamic processes.
