# Supplementary material for: Development and Initial Validation of the Novel Computational Method for Dynamic Intracardiac Blood Flow Evaluation
Source: Diagnostics (Basel). 2026 Apr 30;16(9):1352. doi: 10.3390/diagnostics16091352 (PMC13163574; doi:10.3390/diagnostics16091352)
Supplement: Supplementary file 1 [file diagnostics-16-01352-s001.zip › Supplement S2 (Aims of the study).pdf]

1. Develop a Computer Program: To elaborate a specifically designed Python-based computer program to analyze visual effects of the blood flow motion and turbulence by the rate and degree of blood particle's dynamic fragmentation.

2. Generate Original Flow Visualization: By the program create enhanced dynamic color-coded visualization of intracardiac blood flow using raw radiological data via different imaging modalities.

3. Dynamically Analyze Blood Flow Patterns with newly Proposed Digital Indices: Reveal and distinguish the patterns of LA flow during SR and AF by exploring atrial ICE recordings, digitalize dynamic blood flow changes by calculating TI and BMF indexes, preliminary validate them by correlation with several clinical parameters in the initial cohort of 8 paroxysmal and 8 persistent AF patients undergoing the first RFA procedure.
