# Supplementary material for: Development and Initial Validation of the Novel Computational Method for Dynamic Intracardiac Blood Flow Evaluation
Source: Diagnostics (Basel). 2026 Apr 30;16(9):1352. doi: 10.3390/diagnostics16091352 (PMC13163574; doi:10.3390/diagnostics16091352)
Supplement: Supplementary file 1 [file diagnostics-16-01352-s001.zip › Supplement S3 (isophote level intervals calculations).pdf]

Create isophote level intervals to structure the image.

1. As a first step of processing, there is a need to define isophote level intervals according to the number of the isophote levels. E.g., evenly spaced edges between 0 and 100 over a specified number of 5 intervals were obtained by the formula (Equation S1):

$$Edge(i) = a + i \times \frac{b - a}{n} \qquad \text{(S1)}$$

where:

i – interval index in the range from 0 to n – 1;
n – number of edges;
a – starting value;
b – end value.

2. Five edges were established: 0, 20, 40, 60, 80 and 100. These edges represent percentiles to find the value from pixels within the image's ROI for cardiac cavity. The image pixel represents a whole number that could be positive or negative. All image pixels are collected as separate values, then sorted from the minimum value to the maximum value and later the certain pixels of the certain percentile obtained from an image (Equation S2).

$$C_i = P_{sorted}[i(N - 1) + 1] \qquad \text{(S2)}$$

where:

Ci – the pixel value at the i-th percentile (where i can be 0, 20, 40, 60, 80, or 100);

Psorted – the sorted array of pixel values;

i – the percentile value;

N – the total number of pixel values in Psorted.

3. The intervals were formed based on these percentile values (Equation S3):

$$intervals = \begin{pmatrix} C_0 & C_{20} \\ C_{20} & C_{40} \\ \ldots & \ldots \\ C_{80} & C_{100} \end{pmatrix} \quad \text{(S3)}$$

These intervals are used to define the relation of certain pixel to certain isophote level, assuming that all pixels within a certain percentile range correspond to certain isophote level, starting from 1 and finishing with 5. As a total, 5 isophote levels we defined. E.g., let's assume that the image's ROI has 100 000 pixels. Pixel values within the image's ROI for the cardiac cavity may have a distribution from -200 to 200, with approximately 20 000 pixels having values from -200 to -75 (which represents the 1st interval and corresponds to the 1st isophote level with red color encoding), 20 000 pixels with values from -75 to -25 (representing the 2nd interval and the 2nd isophote level with orange color encoding), 20 000 pixels with values from -25 to 25 (representing the 3rd interval and the 3rd isophote level with white color encoding), 20 000 pixels with values from 25 to 75 (representing the 4th interval and the 4th isophote level with light blue color encoding), and 20 000 pixels with values from 75 to 200 (representing the 5th interval and the 5th isophote level with blue color encoding) (Figure 2d).
