# Supplementary material for: Development and Initial Validation of the Novel Computational Method for Dynamic Intracardiac Blood Flow Evaluation
Source: Diagnostics (Basel). 2026 Apr 30;16(9):1352. doi: 10.3390/diagnostics16091352 (PMC13163574; doi:10.3390/diagnostics16091352)
Supplement: Supplementary file 1 [file diagnostics-16-01352-s001.zip › Supplement S4 (TI calculations).pdf]

TI for an individual isophote.

The TI for an individual isophote is calculated as the ratio of its perimeter to the perimeter of an ideal circle with the same area (Equation S4):

$$TI_{iso} = \frac{P_{iso}}{P_{ideal}} \quad \text{(S4)}$$

where:

TIiso – the turbulence index of an individual isophote;
Piso – the perimeter of the isophote;
Pideal – the perimeter of an ideal circle with the same area as the isophote.

Average TI for an isophote layer.

The average TI for an isophote layer is calculated as (Equation S5):

$$TI_{layer} = \frac{\sum_{i=1}^{n} TI_{iso}}{n} \quad \text{(S5)}$$

where:

TIlayer – the average turbulence index for an isophote layer;
TIiso – the turbulence index of the i-th isophote;
n – the number of isophotes that meet the area threshold.

Average TI for all images per isophote layer

The average TI for all images in a series per isophote layer is (Equation S6):

$$TI_{series} = \frac{\sum_{i=1}^{m} \quad TI_{layer,i}}{m}$$

(S6)

where:

TIseries – the average TI for all images per isophote layer;
TIlayer,i – the average TI for the i-th image's isophote layer;
m – the number of images.

Overall average TI.

The overall average TI is calculated as (Equation S7):

$$TI_{avg} = \frac{\sum_{i=1}^{k} \quad TI_{series,i}}{k}$$

(S7)

where:

TIavg – the overall average turbulence index;
TI series,i – the average TI for the i-th isophote layer across all images;
k – the number of isophote layers.
