# Supplementary material for: Development and Initial Validation of the Novel Computational Method for Dynamic Intracardiac Blood Flow Evaluation
Source: Diagnostics (Basel). 2026 Apr 30;16(9):1352. doi: 10.3390/diagnostics16091352 (PMC13163574; doi:10.3390/diagnostics16091352)
Supplement: Supplementary file 1 [file diagnostics-16-01352-s001.zip › Supplement S5 (BMF calculations).pdf]

BMF calculation

The blood mobility index (BMI) is an aggregated value that is calculated based on the total area of isophotes from all layers in the image that have an area greater or equal to the certain threshold.

1. The area of all isophotes from a layer calculated as a regular sum (Equation S8):

$$A_{lay} = \sum_{i=1}^{j} A_{iso}$$

(S8)

where:

Alay – an area of all isophotes from a layer;
Aiso – an area of a separate isophote;
j – a number of all isophotes.

2. The BMF per image is calculated from the total sum of areas of isophotes from all layers related to the total area of the ROI (Equation S9):

$$BMF_{img} = \frac{\sum_{i=1}^{k} A_{lay}}{A_{ROI}} \times 100$$

(S9)

where:

BMFimg – BMF per image;
k – the number of isophotes layers;
Alay – an area of all isophotes from a layer;
AROI – the total area of the ROI.

3. The average BMF is calculated based on the BMF per image and the total number of images (Equation S10):

$$BMF_{avg} = \frac{\sum_{i=1}^{m} BMF_{img}}{m} \quad \text{(S10)}$$

where:

BMFavg – the average BMF;
BMFimg – BMF per image;
m – the number of images.
