# Supplementary material for: Development and Initial Validation of the Novel Computational Method for Dynamic Intracardiac Blood Flow Evaluation
Source: Diagnostics (Basel). 2026 Apr 30;16(9):1352. doi: 10.3390/diagnostics16091352 (PMC13163574; doi:10.3390/diagnostics16091352)
Supplement: Supplementary file 1 [file diagnostics-16-01352-s001.zip › Supplement S6 (TI and BMF correlations).pdf]

Despite some TI and BMF intracycle variations possibly explained by the 2D mode of recordings, subtle changes in the probe position, noise level and natural blood flow fluctuations, the main patterns of the curves and their interconnection with the visual representation were obviously reproducible within several cardiac cycles, especially in patients with SR without significant structural changes (Figure S7 a-c).

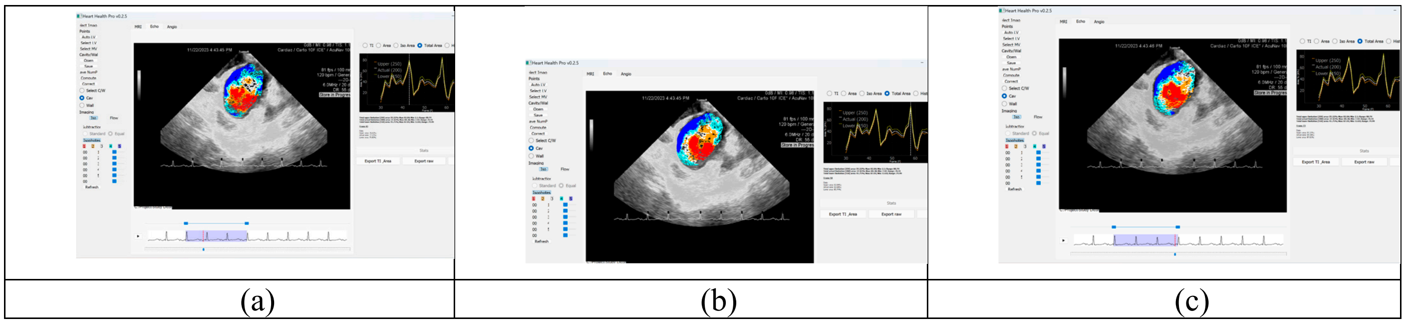

(a) (b) (c)

Figure S7. Comparable color-coded visual representation of BMF (200) peaks of the flow in 3 consecutive cardiac cycles (a, b, c) with exact connection to the same time-moment on ECG (120 bpm, coronary sinus atrial pacing).

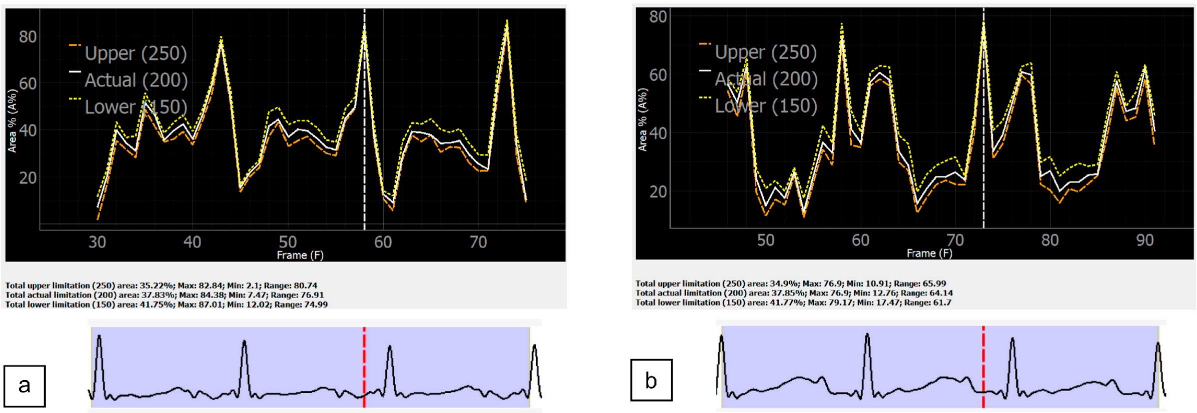

Figure S8. LA BMF curves during fast atrial pacing (120bpm) from CS (a) and low crista terminalis (b); both BMF curves representing peaks occur during LA booster phase but reveal obviously different shapes.

TI and BMF curves were related to transmitral flow timing and correlated with E- and A-wave distributions and amplitudes in patients with SR. The minimal discrepancy between Doppler tracing and the corresponding curves can be explained by consecutive way of recordings with slightly different heart rates (Figure S9 a-f).

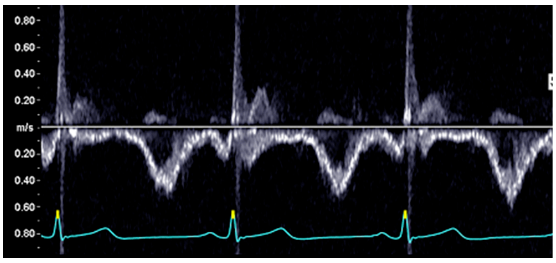

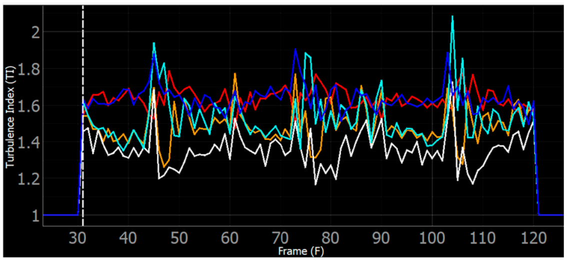

a

b

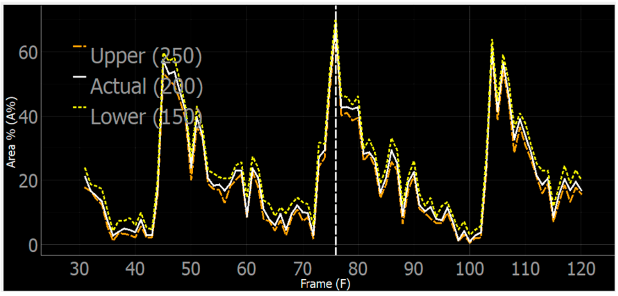

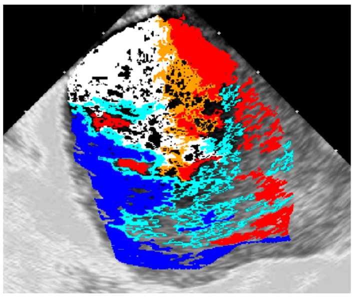

c

d

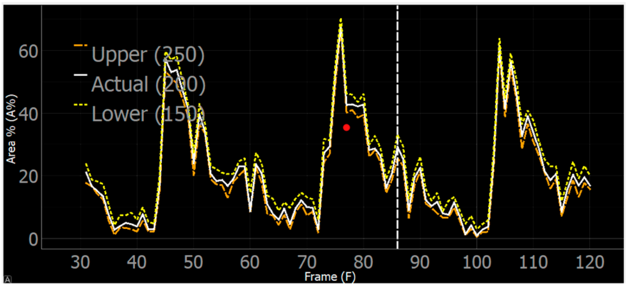

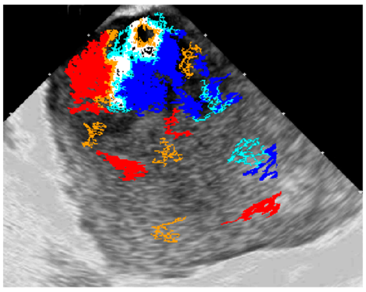

e

f

Figure S9. Mirrored-like correlation of ICE-derived PW Doppler tracing of the transmitral flow with TI (a) and BMF (200) curves (b) during SR in paroxysmal AF patients (c, e); visual

representation of the peak area momentums coincident with the Doppler velocity peaks and associated whole chamber and local vortexes during the conduit and booster phases, respectively (d, f).

Another example of TI and BMF correlation with the doppler (LAA) flow velocities throughout the procedure steps including electrical cardioversion in patient with persistent AF is presented on Figure S10 (a-c).

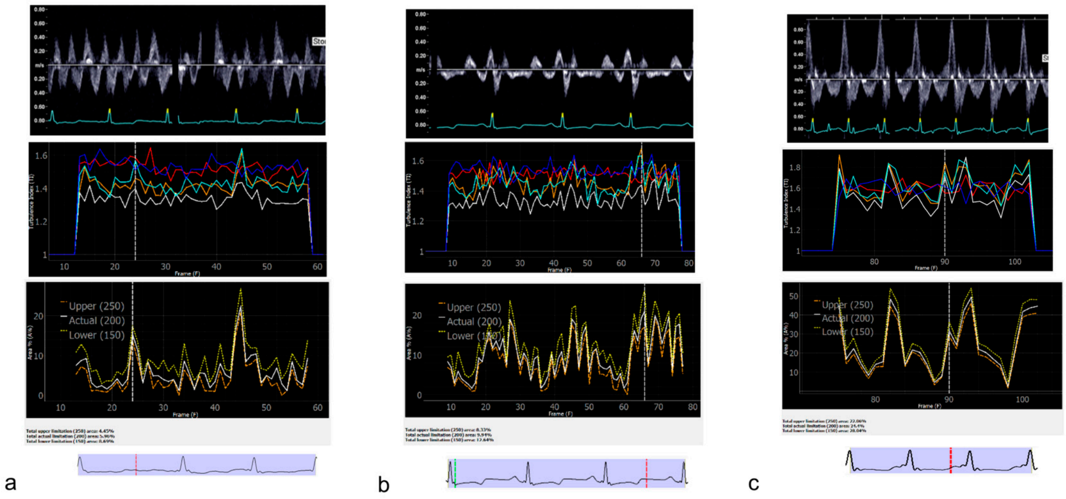

Figure S10. Comparison of TI and BMF in a patient with persistent AF throughout the procedure in connection with LAA velocities: (a) initial AF; (b) SR after electrical cardioversion, (c) elevated SR under isoprenaline infusion.

During AF chaotic blood flow overseen on all levels of flow evaluation with BMF (200) of 6.0% has some significant spikes of TI curves crossing each other (Figure S10a). Despite some degree of the BMF (200) increasing (9.9%) after cardioversion, pattern of the curves looks like very slowly progressing during diastole with even more inconsistent than during AF small amplitude fluctuations of the area. Flow velocities in LAA are also lower than during AF possibly representing immediate LA stunning after cardioversion (Figure S10b). During isoprenaline infusion very close interconnection of TI curves and sharp synchronous changes of different pixels cut-off with dramatic rise of the BMF (200) level (24.4%) with moving of the peaks to the booster phase were obviously correlated with LAA kick velocity acceleration (Figure S10c).

Interconnections between different clinical and instrumental parameters with the flow patterns calculations

Analyzing blood flow patterns showed correlation with several clinical parameters and revealed few tendencies. TI and BMF were interconnected between each other and atrial phases by ECG, LA pressures and blood flow velocities (Figure S11 a-b).

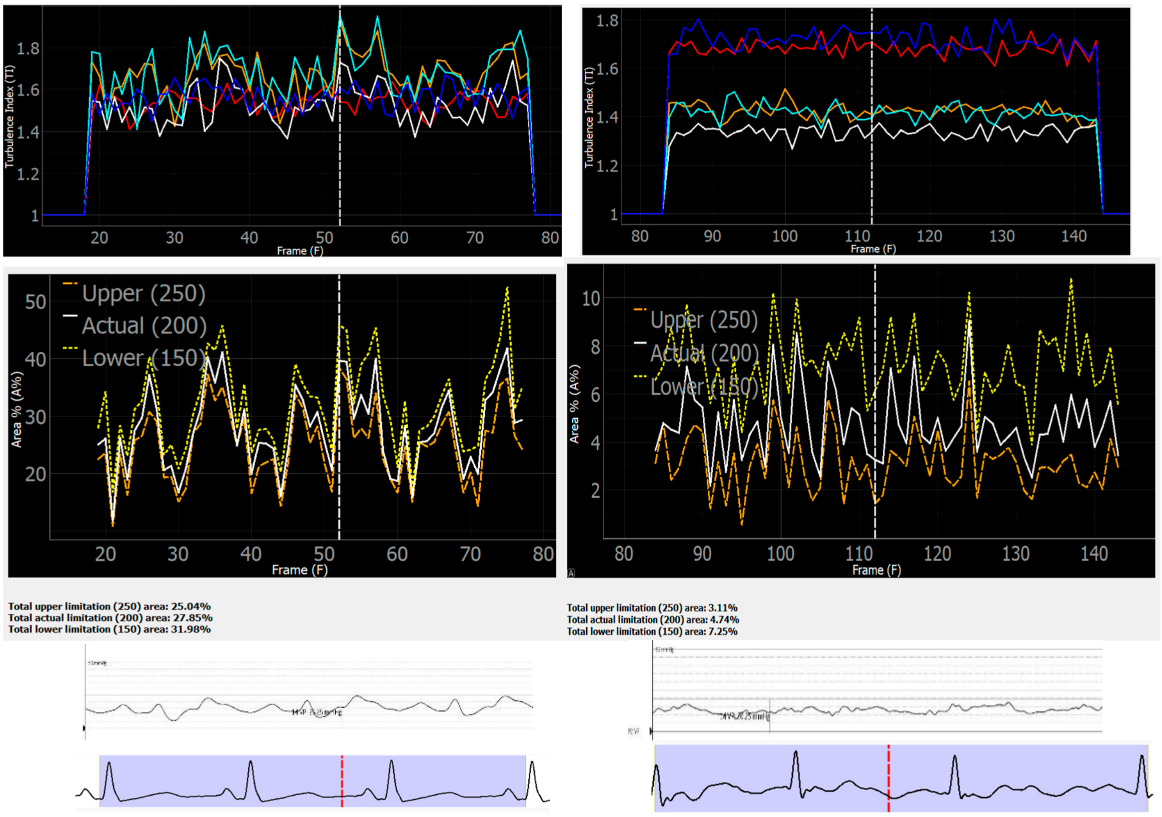

a b

Figure S11. Dynamic interconnections between curves of TI, BMF, LAP and ECG: (a) patient with paroxysmal AF in SR - close correlation between TI and BMF peaks with pressure curve and ECG gating representing three LA cardiac phases; (b) patient with sustained AF- chaotic pattern of TI and BMF curves, weak correlation with LA pressure and ECG; note lower level of average TI (1.52 vs 1.59), BMF(200pxl) (4.7% vs 27.9%) for patient with persistent vs paroxysmal AF in SR, respectively.
