# Supplementary material for: Development and Initial Validation of the Novel Computational Method for Dynamic Intracardiac Blood Flow Evaluation
Source: Diagnostics (Basel). 2026 Apr 30;16(9):1352. doi: 10.3390/diagnostics16091352 (PMC13163574; doi:10.3390/diagnostics16091352)
Supplement: Supplementary file 1 [file diagnostics-16-01352-s001.zip › Supplemental S7 (Final remarks and perspectives)_.pdf]

- In today's era of personalized medicine and advanced technology, a clear understanding of blood flow patterns and the development of new digital indexes for dynamic intracardiac blood flow are essential. These indexes could serve as potential missing elements in various scoring systems and algorithms.
- Achieving optimal blood flow in cardiac chambers, including the LA, likely involves facilitating adequate propulsion with synchronized and variable TI levels across all blood pools, and minimal overall TI during the resting phase. Similar to TI, BMF exhibited significant changes throughout the cardiac cycle, with fewer small fluctuations, and a statistically significant higher percentage of larger particles at their peak and range.
- The proposed indices, particularly BMF, could offer a dynamic alternative to EF. Unlike EF, which is static, BMF reflects continuous data analysis over multiple cardiac cycles and can be further analyzed by phase, providing a more nuanced understanding of cardiac flow dynamics.
- As LA function has been conventionally divided into the three integrated phases: reservoir, conduit and booster, calculation can be made separately for each of them revealing mechanisms of the flow impairment.
- We believe that application of the method for in ventricles can additionally facilitate HF identification, prognosis and treatment.
- Dynamic analysis for all blood pools behaviour could possibly serve as a surrogate for optimal phased potential-kinetic blood energy interchange and equilibrium of the adequate flow. The analogy of "calm, breeze, and tornado" to describe hypo-, normo-, and hyperkinetic atrial flow conditions underscores the need for ongoing research to determine the optimal flow pattern for each patient and to accurately predict future outcomes. Given that turbulence theory is well-established in fields such as aeronautics and weather forecasting, there is a strong potential for its further application in cardiovascular medicine. Finally, the establishment of digital blood flow cardiography could be as simple and useful as the existing golden standard of electrocardiography and proposed indices could finally be presented in simple numerical standard similar to widely adopted ejection fraction.
